# Role and use of evidence in policymaking: an analysis of case studies from the health sector in Nigeria

**DOI:** 10.1186/s12961-015-0049-0

**Published:** 2015-10-24

**Authors:** Obinna Onwujekwe, Nkoli Uguru, Giuliano Russo, Enyi Etiaba, Chinyere Mbachu, Tolib Mirzoev, Benjamin Uzochukwu

**Affiliations:** Department of Health Administration and Management, College of Medicine University of Nigeria, Enugu Campus, Enugu, Nigeria; Department of Preventive Dentistry, College of Medicine University of Nigeria, Enugu Campus, Enugu, Nigeria; Global Health and Tropical Medicine, Instituto de Higiene e Medicina Tropical (IHMT), The Nova University of Lisbon, Lisbon, Portugal; Department of Community Medicine, College of Medicine University of Nigeria, Enugu Campus, Enugu, Nigeria; Nuffield Centre for International Health and Development, Leeds Institute of Health Sciences, University of Leeds, Leeds, UK; Health Policy Research Group, College of Medicine University of Nigeria, Enugu Campus, Enugu, Nigeria

**Keywords:** Evidence, Policy, Policymaking, Role of evidence

## Abstract

**Background:**

Health policymaking is a complex process and analysing the role of evidence is still an evolving area in many low- and middle-income countries. Where evidence is used, it is greatly affected by cognitive and institutional features of the policy process. This paper examines the role of different types of evidence in health policy development in Nigeria.

**Methods:**

The role of evidence was compared between three case studies representing different health policies, namely the (1) integrated maternal neonatal and child health strategy (IMNCH); (2) oral health (OH) policy; and (3) human resource for health (HRH) policy. The data was collected using document reviews and 31 in-depth interviews with key policy actors. Framework Approach was used to analyse the data, aided by NVivo 10 software.

**Results:**

Most respondents perceived evidence to be factual and concrete to support a decision. Evidence was used more if it was perceived to be context-specific, accessible and timely. Low-cost high-impact evidence, such as the Lancet series, was reported to have been used in drafting the IMNCH policy. In the OH and HRH policies, informal evidence such as experts’ experiences and opinions, were reported to have been useful in the policy drafting stage. Both formal and informal evidence were mentioned in the HRH and OH policies, while the development of the IMNCH was revealed to have been informed mainly by more formal evidence. Overall, respondents suggested that formal evidence, such as survey reports and research publications, were most useful in the agenda-setting stage to identify the need for the policy and thus initiating the policy development process. International and local evidence were used to establish the need for a policy and develop policy, and less to develop policy implementation options.

**Conclusion:**

Recognition of the value of different evidence types, combined with structures for generating and using evidence, are likely to enhance evidence-informed health policy development in Nigeria and other similar contexts.

## Background

There is an increasing recognition that strong and effective health systems that are evidence-informed in their operations are necessary to achieve continued improvement in health outcomes in an efficient and equitable manner [1, 2]. The incorporation of relevant high-quality research evidence into the health policy process has been outlined as a key strategy for improving health systems worldwide [3–5]. Thus, evidence-informed decision making has been promoted to aid policy development in most countries [6, 7].

The capability of health systems in low- and middle-income countries to deliver services to the people is severely constrained by polices that are borne out of trial and error rather than evidence [8, 9]. However, policymaking processes do not necessary always follow the clear and straightforward logic of scientific enterprise [10] and some authors have stated that, when evidence is used by policymakers, it is greatly affected by cognitive and institutional features of the political process [11, 12]. Health policymaking, a central element of sustainable health systems, involves a complex process of interactions between policy actors with different powers, interests and agendas [13]. Therefore, ensuring the uptake of evidence for more effective policy and practice is a challenge for health systems strengthening in most low- and middle-income countries [14].

Evidence has been described in the literature as “*what constitutes actual or asserted facts planned for use in support of a conclusion*” [15]. Evidence from research can improve the health policy process, by identifying new issues for the policy agenda, informing decisions about policy content and direction, and evaluating the impact of policy [16–19]. While some authors regard evidence as mostly scientifically-driven facts, others argue that evidence can be formal (such as published research or program monitoring and evaluation) or informal (such as personal experiences, received wisdom and opinions) depending on its process of generation [14, 20, 21].

Analysing the role of evidence in health policymaking is still an evolving area of research. Whereas the focus of previous studies on evidence-informed policymaking has been on the extent to which policies are informed by evidence, there is limited understanding of the relative value of different types of evidence in health policy decisions across different policies and varied contexts.

This paper compares the contributions of different types of evidence in the development of three health policies in Nigeria and explores the contextual influences which affected the utilization of evidence in developing these policies. The results of our study should be of interest and relevance to the key health policy actors (such as policymakers, academia, civil society organizations) who are involved in developing and implementing health policies in Nigeria and similar contexts, and are interested in better understanding and ultimately enhancing the use of evidence in health policy development.

### The Nigerian health system and policymaking

Nigeria operates a three-tier level of healthcare – tertiary, secondary and primary. Provision of healthcare in Nigeria is a concurrent responsibility of the three tiers of government – federal, state and local government. However, because Nigeria operates a mixed economy, private providers of healthcare have a visible role to play in health services delivery [22, 23]. The operational levels have different but sometimes overlapping roles and responsibilities. The Federal Ministry of Health (FMOH) provides tertiary healthcare services as well as technical support to the state and local government health authorities. They also regulate the activities of the lower levels. The State Ministries of Health are statutorily responsible for the provision of secondary healthcare services and the provision of technical support for and regulation of primary healthcare services, while the local governments are responsible for the implementation of primary healthcare and provision of services at the primary care level [24].

In response to the very low ranking of the Nigerian health system in the year 2000 [25], the country embarked on a Health Sector Reform Program (HSRP) in 2003. The Federal Ministry of Health and development partners such as WHO, UNICEF, Partnership for Transforming Health Systems (DFID), and the United Nations Population Fund, among others, initiated and developed different policies and strategies which aim to strengthen and improve the functioning of the health system to become more equitable and efficient in service delivery and consequently improve health outcomes. Three of the key policies and strategies that were developed are the Human Resources for Health (HRH) policy, the Oral Health (OH) policy and the Integrated Maternal, Newborn and Child Health (IMNCH) strategy. These were the subjects of investigation of the present study, which sought to understand the role of evidence in policymaking.

In Nigeria, the FMOH is responsible for policymaking and health policies are made at that level by senior government officials with significant contributions from partners and stakeholders in the public and private sectors. Although the State Ministries of Health can make policies at their level, most polices at the state level are adapted from the national level policies and made to fit the state’s context. A formal process of problem identification, agenda setting, policy formulation, and implementation is used in the policymaking process. However, these stages are usually interwoven with a series of advocacy meetings, stakeholder consultations, and lobbying. The National council on health and the Federal Executive Council must give the final approval on all health-related policies before they can be implemented [24].

The recognition of the usefulness of evidence in policymaking for effective implementation of the country’s HSRP evidence-based policymaking initiatives were established. One such example is the Nigerian Evidence-Based Health Initiative which was developed to inform a plan to support a fair and effective primary healthcare system in Nigeria. However, this program’s focus on only two States limited the uptake of research to inform policy in other parts of the country [26].

Amidst political influence of the ruling party in policymaking, evidence from situation analysis gets used in agenda setting [27]. However, the use of research evidence to inform policy in clinical decision-making in teaching hospitals and in policy implementation in Nigeria has been observed to be very minimal or completely absent [26, 28].

## Methods

This section gives an overview of the study design and background, describing the conceptual framework used in the study and giving a brief description of the three policies and the data collection and analytical methods used.

**Figure 1:**
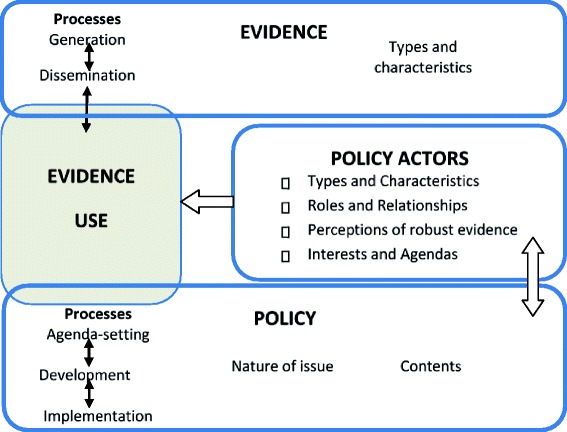
**Framework for assessing the role of evidence in policy development.**

The conceptual framework (Figure 1) has informed the identification of the key information areas for the data collection and analysis in this study. Different types of evidence exist in the literature and they can be grouped into formal and informal [29]. For the purpose of this study, evidence was defined as information – both formal and informal – that can be used in supporting (or otherwise) a conclusion or indicating whether an assumption or proposition is true or valid. Examples of formal evidence include peer-reviewed research reports and health management information system and statistical data, whereas examples of informal evidence include expert knowledge and experiences as well as outcomes of stakeholder consultations. Evidence can be used in decisions either directly (e.g. by indicating effectiveness of a particular intervention within the same or similar context) or indirectly (e.g. by affecting actors’ values and experiences).

As shown in Figure 1, the role of evidence in health policies is perceived as interplay between evidence (i.e. the process of evidence generation, dissemination, and use) and policy processes (agenda setting, development and implementation) [20]. The policy actors determine this interplay through their involvement in evidence and policy processes, affected by their agendas and practices [17, 18]. This interplay is affected by the nature of the policy issue (for example, whether the issue is politically and socially sensitive and/or controversial), the contents of a specific policy (i.e. policy options proposed), and the types and characteristics of evidence available around a specific policy [4, 18]. All of the above occurs within the wider context, which includes different national and international influences [17].

### Study setting

The study was undertaken in Nigeria, a West African country with a population of about 170 million, and conducted at the Federal Capital Territory of Nigeria, where all national policies are made.

### Study design

This was a retrospective cross-sectional qualitative study using a case study approach. Three cases were selected and used to analyse the role of evidence for health policy development in Nigeria. In this study, a case was defined as a health policy or strategy developed for an area of healthcare services (e.g. non-communicable disease, maternal health, and child health) or a component of the health system (e.g. human resources for health).

The cases were purposively selected based on the general criteria that the policy and/or strategy was established within 10 years from the time of the study to enhance recall, and that it was an area of interest to the Ministry of Health. In addition, the policies and/or strategies were selected to represent the following three areas: (1) an area of international prominence (IMNCH strategy, 2007); (2) a neglected area (OH policy, 2012); and (3) an aspect/component of the health system (HRH policy, 2006).

### Description of the policies

#### IMNCH strategy

The Nigerian IMNCH strategy was developed and launched in 2007. Its overall objective is to reduce maternal, newborn, and child morbidity and mortality in line with the millennium development goals (MDGs) 4 and 5. The IMNCH policy was largely a product of a global agenda to improve maternal and child health in the MDGs. The political transition to democracy, accompanied by an improvement in the budgetary allocation to health, provided a window of opportunity to scale-up high impact interventions in maternal and child health in the country [30]. The strategy was developed within the framework of the National HSRP to address the most common conditions responsible for maternal and under-5 mortality in Nigeria. Its development was supported by a Partnership Grant from the Partnership for Maternal, Newborn and Child Health in 2007, in which the FMOH was tasked with the responsibility of coordinating actions and partners to accelerate the reduction in maternal, newborn and child mortality and involved academics, health professionals, civil society organizations (CSOs), and development partners in the policy development processes.

#### OH policy

After several failed attempts in the 1990’s and early 2000’s at developing and obtaining final approval for an OH policy, a National OH Policy was developed and finally adopted in November 2012 in Nigeria. It is intended to achieve optimal OH for at least 50 % of Nigerians through five strategies namely by (1) sustainable awareness creation, (2) early detection and prompt treatment of oral diseases using evidence-based interventions, (3) strategic research, (4) workforce development, and (5) co-ordination of OH activities including institutionalization of modern dental practices. The policy document was developed through multi-stakeholder participation of experts in OH, WHO, and medical practitioners in the three tiers of the health system [31].

#### HRH policy

The HRH policy document was first developed in 2006 by a variety of stakeholders in health made up of public and private sector players following world health reports devoted to addressing the global HRH crisis [31]. An ongoing national health sector reform program also emphasized the poor maternal mortality ratio and under-five mortality rate as well as other anomalies in the health system and was geared towards improving the country’s poor health indices. The policymakers using the national reform and the positive political climate as a platform were motivated to develop the policy to address a number of key challenges in HRH, including planning, recruitment, production, utilization, and retention of health workers.

### Data collection methods

The data was collected between December 2012 and July 2013. Two methods were used to collect data for this study: document review and in-depth interviews.

### Document review

Document reviews were used to identify the different evidence available around a particular policy and inform the development of the initial list of respondents for the study. A total of 27 documents were reviewed, covering all three case studies. Documents included in the review were key policy statements, policy dissemination documents, published consultancy reports, published and unpublished monitoring and evaluation reports, academic publications, research reports, policy briefs, grey literature (e.g. consultancy reports), and health statistics including health management information system reports and country-level publications on websites of the relevant agencies (e.g. FMOH, WHO). In order to retrieve relevant national documents and academic publications, an extensive search was carried out on academic databases (such as PubMed and Cochrane), institution/organization libraries and search websites (such as Google scholar), using key words such as Nigerian health policy, human resources for health policy, integrated maternal and child health policy, and oral health policy. Expert recommendations and citation pearling were also used to identify and retrieve documents.

Once the documents were identified, the executive summary/abstract of each document was reviewed to identify its relevance to the particular policy. The inclusion of documents in the review was guided by relevance of the evidence to the policy issues as perceived by researchers and availability of the document during the timing of the policy development. Relevant information on extent and types of evidence used in policy development and key contextual influences on evidence use was extracted from the documents and summarized according to the key components in the conceptual framework (evidence process and use, context, actors, and policy process). A standardized proforma with these key components was used for the extraction, which was structured around the four components of the conceptual framework.

### In-depth interviews

In-depth interviews were conducted to explore perceptions of usefulness of evidence in policy/strategy development and the key contextual influences on evidence use for policymaking. Detailed information was collected from policy actors such as government policymakers, representatives of CSOs, health workers, development partners, and academics. The list of respondents for interviews was developed using purposive sampling (based on their role in developing health policies and their availability for interview) and informed by review of key policy documents around each of the three case studies, researchers’ knowledge of the actors’ involvement in policy development, and initial meetings with the key stakeholders (as part of consultation in selecting a policy within each case study). The list of respondents was continuously updated throughout data collection using the snowballing technique.

The interviews were guided by a semi-structured question guide focused on respondents’ role and the understanding and perceptions of the role of different types of evidence in health policy development. Most interviews were face-to-face and, in situations where respondents could not be reached within the interview period, telephone interviews were conducted (two interviews in the OH policy case study and one in the IMNCH case study were conducted by telephone). The respondents were assured of confidentiality and anonymity during recruitment. This enabled the respondents to give extensive information on their knowledge, experiences and perceptions on role of evidence in policy formulation.

A total of nine policy actors were interviewed for OH, 10 for IMNCH, and 12 for HRH. A summary of participant types and number interviewed is presented in Table 1. New participants were interviewed until saturation was reached for the respective case studies. Where key actors were not available for interviewing (e.g. retired), their immediate colleagues possessing knowledge of the policy development were approached. All interviews were audio-recorded with informed consent obtained prior to the interview (Table 1).

**Table 1:** **Participant types and number interviewed**

**Participant types**	**IMNCH**	**OH**	**HRH**
Public policymakers and government officials	5	3	5
Academics/Researchers	1	4	1
Professional groups	1	0	1
Civil society organizations	1	1	2
Development partners	2	1	2
Health workers	0	0	1
Total	10	9	12

### Data analysis

A thematic framework analysis was performed, which included stages of familiarization with the data, coding, indexing and charting data, and mapping and interpretation [32]. The findings from the various documents reviewed were synthesized based on the thematic areas and analyzed. All audio recorded interviews were transcribed by the interviewers. A coding tree was used to code the transcriptions according to relevant thematic areas, such as (1) respondents perception of evidence, (2) types and characteristics of evidence, (3) role of different types of evidence, (4) actors, and (5) contextual influences on evidence and policy processes. NVivo10 software was used to aid the data coding and analysis.

### Ethical considerations

Ethics approval for this study was obtained from the University of Nigeria Teaching Hospital ethical review board, before the commencement of the in-depth interviews. Each respondent also gave informed consent before the in-depth interviews were undertaken.

## Results

This section gives a brief description of the policy/strategy development context for the three case studies and highlights similarities and differences between the three case studies in the policy development process. It also presents the types of evidence used in policy/strategy development and the influences of context, respondents’ understanding of the concept, and robustness of evidence on evidence use in development of the policies and strategy.

### Context of policy/strategy development

Based on responses obtained from our interviews, we observed that the three policies were developed amidst different national and international issues. The IMNCH and HRH policies were already familiar and well discussed topics, with previous policies developed. However, the OH policy was regarded as neglected with uncoordinated and haphazard attempts at previous policy developments, which failed after the first OH policy in 1995. All three policies generally followed a formal policy development process which consisted of agenda setting, information gathering, policy formulation, approval, and review and validation. Our respondents’, however, reported that this process did not follow a linear fashion but was iterative and interspersed with stages of lobbying and advocacy, especially with the HRH policy (Table 2).

**Table 2:** **Context of development of the three cases under study (IMNCH, OH, and HRH)**

**IMNCH strategy**	**OH policy**	**HRH policy**
Had high international prominence and was key in most national discourses	Regarded as a neglected area in the health sector of the country	This was a key aspect in most national and international political debates as it cuts across all aspects of health
The need to produce a comprehensive plan of action to address the high maternal and child mortality indices in the country health	This policy was thus developed based on the high oral disease burden in Nigeria	This policy was developed based on the inadequate production, mal-distribution of the available workforce, and the increasing brain drain resulting in shortage of critically needed healthcare professionals
The need for an integrated strategy to promote continuum of care for mother and child	The desire of the relevant stakeholders to standardize practice, organization and delivery of oral health services across the country	
Formal policymaking process (agenda setting to development and final approval of policy)		
Existing strategic framework (national health sector reform plan) was used to guide policy development	No coordinated strategy framework for oral health services	Existing strategic framework (national health sector reform plan) was used to guide policy development
	Failure of previous attempts to develop an oral health policy	

Analysis of interview data revealed that various factors influenced the different stages of policy development. Three factors influenced the agenda setting stage of all three policies: global as well as national considerations and policy framework. The national policy guideline/framework influenced the drafting of IMNCH and HRH policy documents, though this is not the case in the OH policy formulation, where real-life experiences affected the policy drafting and revision stages. In all three policies, and in the OH policy case in particular, both national and global considerations had substantial influences on the probability of policy being approved. The international clamour for the need of an OH policy by global actors, for example by WHO, and the persistence of one specific national actor, are all factors that were found to have influenced the likelihood of policy approval.

### Respondents’ perception of evidence

Across the three case studies, participants perceived evidence processes as a formal process of gathering information to inform decisions. However, different stakeholders appeared to adopt diverse and often conflicting views of what constitutes evidence, as their understanding of the concept seemed to reflect their personal experiences. We did not identify a single dominant common definition of ‘evidence’, but rather a plurality of interpretations, but this was not highlighted as an issue of concern by the interviewees. In some cases, perceptions of evidence were broad and in others it was linked synonymously with research, albeit with less certainty about the meaning of the word ‘evidence’. Most respondents gave a definition of evidence which is synonymous with research.

“*My understanding of evidence use is having concrete data that has been collected through a process that is considered legitimate and you give reference to that data. It could have been a survey.*” (HRH, CSO; OH, Academia)

However, a minority of respondents also gave a broader definition of evidence, suggesting that this can also include informal types:

“*Evidence is like a mere idea that have been harvested and shows where you are, and informs what you intend to subsequently do. In this context, it should prove there is a situation*.” (HRH, Policymaker)

### Types of evidence used

Ten types of evidence were perceived by the respondents as being used to develop policies within all three case studies. These are survey reports, research publications, national and international policy documents, systemic review reports of programs, proceedings from expert consultation meetings, experience and opinions of experts and policymakers, epidemiological reports and documents on lessons learned from international experiences, and best practice guidelines (Table 3).

**Table 3:** **Types of evidence used across the three case studies**

**Types of evidence used**	**Case studies**		
	**IMNCH**	**OH**	**HRH**
Survey reports, situation analysis, national data sets, institution data sets	✓	✓	✓
Research publications (international and national)	✓	✓	✓
Existing policy documents (national)	✓	X	✓
Systematic review reports	✓	X	X
Epidemiological reports	✓	X	X
Proceedings of expert consultation meetings	✓	✓	✓
Lessons from international experience and best practice guidelines (policies and publications)	✓	✓	X
Health management and information system data	X	X	X
Monitoring and evaluation reports	X	X	X
Expert and policymaker opinions and experiences	✓	✓	✓

Both formal and informal types of evidence informed the development of the three policies, though our analysis shows that the formal types of evidence played a larger role. The quote below depicts this statement.

“*Basically, evidence from program implementation, research findings, program reports, I think this are for me the most important.*” (IMNCH, Policymaker)

As shown in Table 3, formal evidence (national and international) played a more prominent role in the development of all three policies. Informal evidence seemed to play a role in HRH and OH policies more than in IMNCH policy.

Amongst all evidence used, the one considered to be the most important by the majority of respondents across the three cases were findings from national surveys (such as baseline surveys or situation analysis) because they were context-specific, timely and gave a true picture of what was on the ground. Most respondents felt that the methodological rigor, availability of survey reports, relevance, and ease of obtaining the information from these surveys were also what made it especially useful.

“*The evidences we had were based on research activities and surveys which are national in their scope, and then …… they were quite extensive and rigorous*” (OH, Academia).

However, there were a few disparate views as regards labelling this singular type of evidence as the most important, because according to the respondents, “*all types of evidence generated complements each other, we cannot say one is the best*” (HRH, Academia). For example, in the HRH case study, evidence obtained from opinions and experiences of experts in the field were considered important by some respondents, especially the policymakers. They claimed that this form of evidence helped to augment the findings from the baseline survey. In IMNCH, published research findings, especially those from a series of publications showing evidence of high impact interventions for maternal and child survival published in the *Lancet* [33], were important by giving an insight into best practices for maternal and child health issues and added to the body of evidence in developing the policy.

“*For me the ones I thought were most important was evidence of effectiveness of high impact intervention from the Bellagio* [Lancet [33]] *survival series*” (IMNCH, Policymaker).

An analysis of the various responses highlight the use of a wide breath of evidence-informed policies, which included both formal and informal evidence. The nature of methodology (rigorous or not) informed the likelihood of evidence.

“*Evidence am looking more at empirical data….empirical data. But beyond looking at empirical data, I am also interested in the process of gathering that data.*” (DP, CSO)

In response to our questions on what evidence the respondents consider as robust, we documented eight characteristics of robust evidence as perceived by our respondents, namely quality/accuracy, credibility, relevance, accessibility, comprehensiveness, context specificity, of national scale, and representativeness of evidence. The following quotes substantiate these findings:

“*My understanding of evidence use is having concrete data that has been collected through a process that is considered legitimate and you give reference to that data. It could have been a survey. I mean it’s a fact that figures don’t lie. --- figures about distribution, retention, rural–urban disparity and all that stuff.*” (HRH, CSO)

“*If you are writing on a national document then you use data that is representative of the whole country*.” (IMNCH, Policymaker).

The issue of comprehensiveness of evidence was emphasized only by the respondents in the IMNCH case study and evidence being of national scale and representativeness was emphasized in the development of the OH policy. The importance of all other characteristics of evidence was shared by respondents across the three policies.

### Role played by different types of evidence in policy development across case studies

The various types of evidence mentioned previously informed different stages of the policy development process in the three case studies (Table 4). Survey reports and research publications informed the policy development process across the case studies. These survey reports and research publications were most useful in the agenda setting stage because they provided the push needed to bring the topic on the policy agenda thus initiating the first stage of the policy development process.

**Table 4:** **Role played by different types of evidence in three case studies**

**Stages of policy development**	**Evidence used**		
	**IMNCH policy**	**OH policy**	**HRH policy**
Information gathering	National survey reports, institutional data, epidemiological reports, research publications, aggregated data from states	Institutional data, research publications, lessons learnt from international experience	Institutional data, situation analysis reports, research publications
Agenda setting	Publications of best practices, e.g. *Lancet* series, systematic review reports, research publications	WHO publications, national survey reports, research publications, publications of best practices	Situation analysis reports, research publications
Development of policy draft	Expert consultation reports, existing policy documents	Experiences and opinions of experts, expert consultation reports	Expert consultation reports, experiences and opinions of experts, existing policy documents
Review of policy draft	Expert consultation reports, synthesis of previously collected information	Expert consultation reports, synthesis of previously collected information	Expert consultation reports, synthesis of previously collected information
Approval of policy document	Expert consultation reports	Expert consultation reports	Expert consultation reports

“*Well, the survey found many discrepancies in the ratio of health workforce in relation to all health sectors. It was terrible. When the data was presented we shuddered. When we saw from literature review what other countries are doing, we were challenged to do something*” (HRH, Policymaker)

In addition, journal publications from the *Lancet* series in IMNCH policy [33] and WHO publications in OH policy [34] were used to a large extent in the problem definition stage and helped buttress the data obtained from the surveys. They provided data on best practices for the OH and IMNCH policies, which helped in development of the policy (Table 4).

On the other hand, reports from expert consultations were perceived by the respondents as useful in the policy drafting stage because they now built on the information provided by the surveys to improve the body of evidence used. The expert consultations brought in a practical aspect to the policy development because most of the evidence generated from this source was based on hands-on experience of the stakeholders, which added value to the evidence obtained, as illustrated below:

“*What happened was there was a stakeholders meeting and we got a lot of information from them, sat down together to brain storm on the information and that was what was utilized in the final writing*” (OH, Policymaker)

The situation analysis and survey reports were useful to different actors for different reasons: to the academics, these types of evidence gave a credible idea of the situation and provided guidance to the direction of their technical input; the government policymakers found them useful in providing information that was representative of the country and context appropriate; to the development partners, they provided useful information to guide decisions of resource allocation and technical input; and, finally, the CSO’s found these useful for providing evidence of current health situations in terms of figures and numbers that would enable more effective lobbying.

### Key influences on evidence use

We found that global considerations, existing policy guidelines, and burden of health needs were the major contextual influences which helped set the agenda for the three health policies. The utilization of evidence in each of these three policies was influenced by different factors from these three broad areas (Table 5).

**Table 5:** **Influence of context on evidence use**

**Factors**	**IMNCH**	**OH**	**HRH**
Global considerations/movement	WHO partnership for maternal, newborn and child health movement provided support for partner countries to develop an IMNCH strategy	There was an international push towards development of oral health policies in Africa	Inability to meet the international standard for health worker to population ratio
			International move to resolve HRH crisis especially in Africa
Existing policy/guideline/frame work	Prior existence of the reproductive health policy and child health policy	Fragmented drafts of previously written policies which had not been adopted	Existence of health sector reform plan which emphasized the need for equitable distribution and health worker retention
	Existence of implementation strategy for the child health policy	This was the first OH policy adopted in Nov 2012	
National considerations	Ongoing National health sector reform plan which showed areas of need	Presence of policy champion	Policy champion and ongoing health sector reform plan

A general global push towards evidence-based practice was identified by the respondents as an important contextual influence which increased the use of evidence in all three policies. For example, the international push for the development of the OH policy was predominantly from WHO, which had directed its African region countries to all develop an OH policy as part of their regional OH strategy [35].

“*The WHO directive and with Nigeria being a major stake holder in the WHO, I think that facilitated the approval for developing of Oral Health Policy in the country.*” (OH, Academia).

All four previous attempts at formulating an OH policy in Nigeria in the past three decades were unsuccessful. The first three attempts (between 1984 and 1999) failed because of an unfavourable political environment during military rule, whereas a fourth attempt at developing an OH policy (2004–2009) failed due to non-observance of national stipulations for policy development [36].

## Discussion

Multiple evidence types were used in the development of three health policies in Nigeria. Policymakers and other actors, though guided by their perceptions of robust evidence to make decisions on which evidence to use, were also perceived importance of experiences and expert opinions possibly giving room for a ‘non-scientific’ angle to policymaking. Eight characteristics of robust evidence were perceived to be important by policy actors in all policies. These qualities were attributed to nationally-generated evidence, such as reports of surveys, and other datasets generated through government machinery. Context was an important determinant of perceived characteristic of robust evidence. The fact that methodological rigor was mentioned by some policymakers as a characteristic of robust evidence suggests that these individuals have some understanding of what constitutes methodological rigor and this degree of understanding reflects their background.

Different stakeholders seemed to adopt diverse definitions of what represents evidence – possibly influenced by their personal experiences in policy development. Although the respondents never identified this lack of a shared definition of evidence as an issue of concern, it is clear that an unresolved tension existed among our informants, as people kept referring to different concepts and attributes of what represents evidence for and about policy.

The actors’ understandings of evidence appear also to have influenced which evidence was used. The fact that most respondents defined evidence as a formal entity where legitimate and concrete data are collected and processed could be a reflection of why formal types of evidence (research and survey data and reports, etc.) were used most frequently in setting the agenda. For example, in the OH and HRH case studies, the use of situation analysis reports, survey reports, and published research confirmed this potential relationship between perception of what constitutes evidence and its use. This is also true for IMNCH, where evidence of best practices from international publications constituted the main form of evidence used. In addition, it could be that the actors’ beliefs had been pre-shaped by the public policymaking structure in the country which seems to rely on documented evidence to be used to either make a point or influence a policy decision. This finding is similar to what Sutcliffe and Court reported [37], namely that actors’ ideologies, principles and political orientation ultimately affect how and what types of evidence get used for policymaking. This became more obvious in our study because the perception of the government policymakers, “*who were the drivers of the three policies*”, was that stakeholders’ experiences were a very important form of evidence which led to its use in the OH and HRH policy formulation.

In literature, evidence-informed policy was defined as public policy informed by rigorously tested established evidence in the design, implementation and refinement of policy [38]. Our study shows that a wide breadth of evidence was used in formulating the three policies in Nigeria. However, more emphasis was laid on the formal type of evidence and most evidence was used at the agenda-setting and less during the development stage. This could be due to the fact that Nigeria has a documented process for policy development as shown by the different strategic frameworks and the Nigerian constitution (FMOH) in addition to the international drive towards evidence-based policymaking.

Similar types of evidence were generated to formulate the three policies. This suggests that the same type of evidence is generally accessible to those formulating policies in the country or there is limited knowledge about the various types of evidence that are available and possibly gathered for policymaking. There may therefore be a need to enlighten those involved in policymaking about what constitutes evidence so as to broaden the scope of types of evidence used for future policymaking.

Evidence use for policy agenda setting appeared to be extensive and adequate in all three cases and the situation analysis and survey reports were useful to different actors for different reasons. Our study revealed that evidence was used as input to decision-making for technical experts, conceptualizing the situation for government policymakers, implementing partners and CSOs, and persuading actors to buy in for certain policies, all of which was achieved by CSO lobbying. This is similar to findings by Shadish et al. [39], who also revealed that evidence could be used for three purposes – instrumental (input to decisions making), conceptual (helping towards a better understanding of the subject matter), and strategic (to persuade other actors as the means to attain a specific goal).

Formal evidence, such as research findings and data sets, were used in agenda setting, because of their context relevance, rigour and timeliness. The ease and availability of obtaining information from these sources of evidence also played a role in their use for policy/strategy making. According to Shaxson [40], some key characteristics of evidence which influence whether it is used include quality/accuracy, credibility, relevance, and accessibility to policymakers. In addition to these characteristics, comprehensiveness, context relevance, and representativeness of evidence were considered characteristics of robust evidence in our study.

Formal evidence, like research and survey and program reports, were used in policy development, while there was little or no mention of other types of formal evidence such as health management information system data. This could be attributed to their perceived unavailability. Thus, only evidence considered reliable or accessible and truly reflecting which evidence the policy actors felt was the best was used to establish the need for policy development.

The informal type of evidence used, which was the expert consultation meetings, emphasized the fact that evidence was subject to what the actors considered important and relevant and also what they perceived evidence to be. The fact that some respondents – such as influential government policymakers – felt this form of evidence was very important, was synonymous with its use in the drafting and writing up of the policy, even though other stakeholders, like academics and CSO’s, felt less strongly about this form of evidence. However, this use of evidence is consistent with what was reported in previous studies [20], which observed that apart from formal documented evidence from data and research, experience of various stakeholders and decision makers can also be influential in the policy development process.

Contextual factors influenced policy formulation and evidence use for the three case studies, albeit differently. For instance, the interests of international partners and agencies gave the initial push for prioritization of maternal and child health on the national agenda, and the subsequent development of the IMNCH strategy. Whereas the need to develop an OH policy was underscored by the absence of a strategic framework for OH services and previous failed attempts at policy formulation, rather than an international push. National surveys and empirical studies that clearly identified health problems, policy gaps and bottlenecks, and made recommendations of strategies or guidelines for implementation, provided useful guidance during the process of drafting policy contents. A central influence on evidence process across case studies in Nigeria was the recognition of the global movement on evidence-based best practices. Although somewhat different in its nature to international treaties such as the MDGs, this movement triggered a national awareness of the need to develop comprehensive, evidence-informed policies and plans of action. Though similar to what was found in India as part of the wider study, evidence use is being progressively recognized as a highly contingent process that varies across settings and time [41, 42]. In this sense, context can be seen as a potential determinant of the use of evidence.

The reliance on international evidence for providing new policy ideas, in this case, may be linked to the strategic role policy actors (e.g. WHO) played in identifying and disseminating evidence for informing the policy process. A wider range of influences were identified at the agenda-setting stage than during policy formulation and approval. This is similar to what other authors found [20]. Similar to the above, multiple influences, often comprising international and national factors, can ensure that a policy is finally approved, as was the case with the successful approval of OH policy following several previous attempts. On the other hand, fewer factors affected policy formulation, though a clear guideline or a framework can mainstream policy processes and can potentially provide a framework for ensuring actors’ participation and possibly addressing any discourse (such as different views of robust evidence).

Our findings suggest that recognition of the value of formal and informal types of evidence, combined with structures for generating and using evidence, are likely to facilitate generation of evidence for policy development. Evidence that is easily accessible to policymakers and is in a user-friendly form, will most likely be used in policy development. Furthermore, involvement of technical experts (including researchers) who have an appreciation for evidence and its use in policymaking, also contributes to evidence-based policymaking.

Several potential implications for policy and practice emerge from our study, which should be of relevance to the policy actors interested in improving the role of evidence in health policymaking in Nigeria and other similar contexts. First, different policy actors need to be aware of the understanding of the concept of evidence by others, including their preferences for robust evidence. In the longer term, different policy actors can work towards developing a shared understanding of robust evidence. Secondly, the knowledge of different types of evidence and their importance would enhance the use of both formal and informal types of evidence which should improve the quality of evidence generated for policy. Thirdly, a decentralized consultative approach using a variety of mechanisms to obtain wider participation and input from a range of stakeholders at all levels, including national and sub-national, government and non-government, as well as the public groups. These mechanisms include consultations with multi-stakeholder groups and expert working group meetings.

A possible limitation of the study is that we relied mostly on the understanding of the role of evidence as perceived by the policy actors as this creates the potential for recall bias. Whilst we attempted to look for signs of evidence use in policy documents (e.g. references to specific studies), the systematic comparison of available versus the used evidence was outside the scope of this study.

## Conclusion

Different types of evidence were used to formulate policy in the three case studies. Most evidence generated was used to set the agenda and policy discourse. Apart from the IMNCH strategy where evidence was used to select interventions, we did not find any indication that suggest evidence was used to determine policy options (‘evidence on policy’) for the OH and HRH policies.

Relevance, comprehensiveness, availability, context-appropriateness, affordability, methodological rigor, timeliness, and representation of country situation were characteristics of robust evidence as perceived by policymakers. Although it is unlikely that these characteristics of robust evidence would be met at the same time, research organizations and academics in the country are challenged with the need to generate evidence that meet these criteria. Policymakers and influencers, on the other hand, need to exercise some judgment about priorities and preferences in the selection of available evidence for policymaking.

### Availability of supporting data

Study instruments and data are available on request from the corresponding author.

## Abbreviations

CSO: Civil Society Organisation
FMOH: Federal Ministry of Health
HRH: Human resources for health
HSRP: Health Sector Reform Program
IMNCH: Integrated maternal neonatal and child health
MDG: Millennium Development Goal
OH: Oral health
PMNCH: Partnership for Maternal, Newborn and Child Health
